# Impact of Thermophysical Properties of High-Alloy Tool Steels on Their Performance in Re-Purposing Applications

**DOI:** 10.3390/ma15238702

**Published:** 2022-12-06

**Authors:** Aaron Berger, Santiago Benito, Philipp Kronenberg, Sebastian Weber

**Affiliations:** 1 Chair of Materials Technology, Institute for Materials, Ruhr-University Bochum, Universitätsstrasse 150, 44801 Bochum, Germany; 2 Chair of New Manufacturing Technologies and Materials, University of Wuppertal, Bahnhofstrasse 15, 42651 Solingen, Germany

**Keywords:** thermophysical properties, thermal conductivity, thermal diffusivity, tool steels, electronic thermal conductivity, repurpose, circular economy

## Abstract

Resource efficiency and circularity in the context of sustainability are rapidly gaining importance in the steel industry. One concept regarding circular economy is “repurposing”. In the context of this work, worn-out machine circular knives are used to produce new chisels for woodturning. The chisels can be extracted parallel or perpendicular to the rolling direction of the primary production process, resulting in an associated carbide orientation of the repurposed tool. The rolling direction, and therefore carbide alignment, will influence the wear resistance and the thermophysical properties, whereby the thermal conductivity will determine the temperatures at the tip of the chisel. Therefore, the thermal conductivity was investigated with the dynamic measurement method, where the specific heat capacity, density and thermal diffusivity of the extracted chisels and industrial reference chisels were measured separately. Moreover, the electrical resistivity was measured in order to calculate the electronic thermal conductivity according to the Wiedemann–Franz–Lorenz law. It was shown that all of these parameters exhibited different degrees of variability with rising temperature. In a detailed analysis, the thermal diffusivity could be identified as an essential parameter of thermal conductivity. By taking two conventional chisels with different chemical compositions and heat treatments into account, it can be seen that the microstructure determines the thermophysical properties. Considering the carbide direction, the chisels that were extracted parallel to the rolling direction showed differing thermophysical properties. Therefore, the carbide orientation is shown to play a significant role regarding the heat dissipation at the cutting edge, because differences, especially in the electronic thermal conductivity in the parallel and perpendicular extracted chisels, can be measured. In addition to the wear resistance factor, the thermal conductivity factor now also supports the removal of the repurposed chisels parallel to the rolling direction.

## 1. Introduction

Steel is a universally applicable material and therefore essential for today’s economy and society; for instance, the raw steel production figures have doubled in the last 20 years [1]. With regard to sustainability, steel exhibits a high potential for resource efficiency because of its durability and a recyclability of nearly 100%. Nevertheless, the recycling of steels rarely achieves this efficiency due to contaminations even in remelting in basic oxygen or electric arc furnaces [2]. In addition, this type of recycling produces greenhouse gases, which must be considered as well. 

According to Potting et al. [3], three different groups of the so-called 10 R-list can be identified in terms of the degree of circularity. The “Recycling” and “Recover” approaches are in the group with the lowest degree of circularity, whereas high degrees are preferable. The highest degrees of circularity can be achieved by the strategies “Refuse”, “Rethink” and “Reduce”. Classified in group two are the strategies “Re-use”, “Repair”, “Refurbish”, “Remanufacture” and “Repurpose”.

The strategy used here belongs to the “Repurpose” category, in which an old and no-longer-usable product is converted into another product with a different application [3]. This enables the use of raw materials within an extended life cycle, improving the carbon footprint massively. Different approaches have been studied in the literature regarding different industries; for example, the cement industry [4] or even the production of mineral wool [5]. In this work, the concept of “Repurpose” is applied to worn-out machine circular knives used in paper-cutting processes. Blanks for woodturning chisels were taken out of the circular knives and implemented in the conventional production process in order to maximize the material efficiency [6]. The manufacturing process of the machine circular knife determines the microstructure—or, more specifically, the carbide orientation—of the woodturning chisels. The chisels can be taken out along or across to the primary rolling direction [7]. As examined in previous works, the relative general orientation of these microstructural constituents influences critical macroscopical properties related to their system-related performance, such as abrasive wear resistance and maximum service temperature [8,9,10].

The service life of the extracted chisel depends mainly on the wear during woodworking. Wear describes the progressive loss of material from the surface of the base body in a tribological system, whereby the tribological system is determined by the base body in a relative motion to a counter body, sometimes in combination with an intermediate medium [11,12]. Therefore, the wear resistance is not a material property, but a property of a specific material in the given tribological system [11,12].

When relative motions in a tribological system occur, heat results from the energy lost due to friction between the base and the counter body. Due to the high rotational speed and the high thrust in woodworking, high temperatures are therefore generated at the cutting edge, which as a result is a decisive factor for the material selection.

By combining these factors, the chisel underlies a coupled thermomechanical problem. Besides the wear resistance, the thermal conductivity is a further governing parameter which determines the temperature at the cutting edge. Higher conductivities are favourable because of an improved heat dissipation off the cutting edge. Furthermore, both parameters are strongly temperature-dependent and have to be taken into account when considering a tribological system, especially when a higher thermal conductivity lowers the operating temperature and thus results in a higher hardness and increased wear resistance during operation [10,13]. The hardness and wear resistance is furthermore strongly dependent on the temperature, making this factor even more relevant [13,14]. The thermal conductivity is determined by the chemical composition, as well as the heat treatment and the resulting microstructure [8,9,10,15]. In particular, the carbide morphology and orientation, as well as the solution state of carbon in tool steels, plays a significant role in the thermal conductivity [8,9,10]. 

In the scope of this work, the thermophysical properties of the repurposed woodturning chisel made out of a lederburitic cold work tool steel will be described and evaluated by comparing them with those of a conventional chisel made of a carbon martensitic tool steel and a performance chisel made of a high-speed steel as benchmark materials. In particular, the repurposed chisel can be taken out across and along the rolling direction, a decision which might influence the thermal conductivity and thus has to be studied. In particular, the influence of the carbide morphology regarding the chisel extraction, as well as the influence of the tempering behavior of the investigated alloys on the thermophysical properties, is of special interest. Due to technological challenges in the sample extraction campaign, this work investigates the thermal conductivity by two independent routes: namely, using the dynamic indirect measurement method and the Wiedemann–Franz–Lorenz law to shed light onto its the electronic component [16]. In all cases, the temperature dependency was considered.

## 2. Materials and Methods

### 2.1. Materials and Metallography

**Figure 1:**
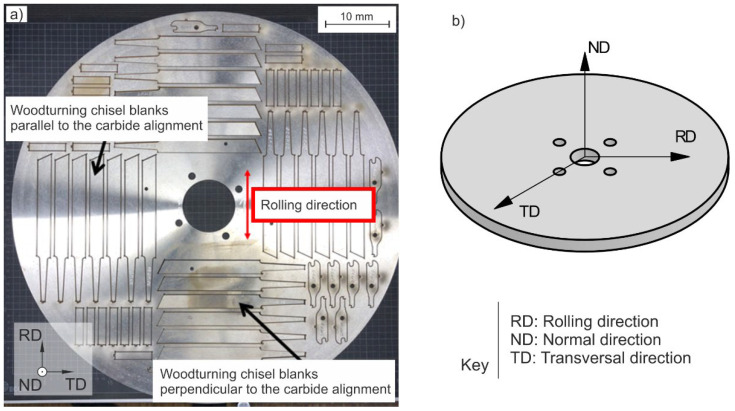
(**a**) Worn-out machine circular knife with repurposed chisel blanks, taken out in the rolling and transversal directions (RD, TD, respectively). (**b**) Schematic diagram of the axes system selected.

Repurposed woodturning chisel blanks were taken out of the worn-out machine circular knives parallel and perpendicular to the carbide direction by laser cutting; the analogue of the primary hot-rolling and its transversal direction (Figure 1a,b) shows the selected axis system. Throughout this work, reference to the specimen or micrography orientation will be made using the following nomenclature: rolling direction (RD), transversal direction (TD), and normal direction (ND).

The blanks were then manufactured into ready-to-use chisel shapes “A 20” according to DIN 5144 [17]. Benchmark chisels are also considered as a standard version according to this shape. The repurposed chisels were made of the cold work steel D2, and the benchmark chisels were made of the tool steel L2 and the high-speed steel M2, respectively. The corresponding chemical compositions are shown in Table 1. Optical emission spectrometry was carried out to determine the chemical composition with an emission spectrometer from OBLF GmbH, Witten, Germany. 

All materials were produced by casting, hot forming and a subsequent heat treatment, consisting of hardening and tempering according to industrial conditions (Table 2).

For metallographic investigations, samples were cut from the three chisels. The samples were ground with SiC paper and were subsequently polished with a diamond suspension with a mean grain size of 6, 3 and 1 µm.

Microscopic images were obtained with a Vega 3 SHB SEM by TESCAN ORSAY HOLDING, a. s, Brno, Czech Republic, using secondary electron contrast with a voltage of 20 kV and a working distance of 10 mm. 

### 2.2. Thermophysical Properties

#### 2.2.1. Thermal Conductivity

The thermal conductivity λ was first determined by the dynamic indirect measurement method, whereby the separate and temperature-dependent parameters of specific heat capacity c_p_, thermal diffusivity a and density ρ are measured in a temperature range of room temperature up to 500 °C (Equation (1)) [16,18]:

λ = c_p_·a·ρ (1)

The specific isobar heat capacity c_p_ was determined using a differential scanning calorimeter type HDSC PT-1600 by Linseis Messgeraete GmbH, Selb, Germany. After stabilizing, two empty crucibles were measured for baseline correction. Calibration with a sapphire specimen in one crucible was performed immediately afterwards. Samples with a diameter of 4 mm and a thickness of 0.5 mm were ground by mesh 100 SiC paper to ensure a good heat transfer between specimen and crucible. They replaced the sapphire after calibration. Heating remained unchanged between baseline, calibration and sample measurements. The program consisted of a linear heating rate of 20 K/min up to 500 °C, a dwell time of 1 min and cooling to room temperature. Three independent test series were carried out for each investigated material.

The density was measured at room temperature using Archimedes’ principle. Measurements were performed in air and ethanol, subsequently, using a high-precision laboratory balance CPA 225D by Sartorius AG. The room-temperature density of all three samples was determined in a series of five different measurements. Temperature dependency was determined by combination with the thermal expansion coefficient α_th_, which were measured using a vertical dilatometer with an Al_2_O_3_ differential measurement system type L75 Platinum Series by Linseis Messgeraete GmbH, Selb, Germany. Round samples with a diameter of 4 mm and a length of 20 mm were investigated by means of dilatometry. Reference parameters were the room temperature as well as the length of the sample at room temperature. Three independent measurements on three separate specimens were carried out for each material and temperature. The average and the standard deviation were then calculated.

The thermal diffusivity was measured by the laser-flash principle using a laser-flash analyzer type LFA 1250 by Linseis Messgeraete GmbH, Selb, Germany. The samples with a diameter of 10 mm and a thickness L of 2 mm were ground with 1000 mesh SiC paper and coated with a thin graphite layer to maximize the absorption of the laser radiation to ensure a precise measurement. The sample upper surface temperature was constantly monitored during laser irradiation by an infrared sensor. Thermal diffusivity was determined by the t_1/2_ time, which is the required time for the upper side of the specimen to reach 50% of its maximal temperature (Equation (2)) [19]:

a = 1.38·L^2^/(π^2^·t_1/2_) (2)

At least three independent measurements on three separate specimens were executed for each material and temperature. The average thermal conductivity according to Equation (1) was calculated using their mean values. The standard deviation of the thermal conductivity was then calculated according to the Gaussian rule of error propagation.

Unfortunately, the extraction of the laser-flash specimens was not possible in the rolling (RD) and transversal directions (TD) due to the required geometry being at odds with the knife thickness (the thickness was less than the required sample diameter of 10 mm, Figure 1). Instead, the laser-flash samples were cut out in the normal direction of the disks (ND) (Figure 1). Thus, a direct comparison of the thermal conductivity in the orientations of interest was not possible. The next subsection outlines the alternative approach that makes a quantitative evaluation possible.

#### 2.2.2. Electrical Resistivity

Resistivity and Seebeck coefficient measurements were performed by the four-wire measuring method using an LSR-3 system by Linseis Messgeraete GmbH, Selb, Germany. Cylindrical samples with a diameter of 4 mm and a length of 10 mm were used. The thermal gradient of 50 °C was applied by a secondary heating source on the bottom of the sample. The probe current was applied with a magnitude of 100 mA. Three independent measurements on three separate specimens were carried out for each material and temperature. The average and the standard deviation were then calculated.

The thermal conductivity consists of different components, namely a phononic contribution by lattice vibrations, an electronic, and a magnonic contribution, these being independent of each other [18,20,21]. The magnonic contribution is particularly small and can be neglected in this case [21]. Thereby, a calculation of the resulting thermal conductivity was obtained according to Equation (3):

λ_res_ = λ_electronic_ + λ_phononic_ (3)

The electronic contribution can be calculated using the Wiedemann–Franz–Lorenz law [16,22]:

λ_elelectronic_ = T/σ·(L_0_ − S^2^), (4)

where the S is the Seebeck coefficient, σ is the electrical conductivity, and L_0_ is the known Lorenz number.

The consideration of the electronic part enables the differentiation and influence of the parallel and perpendicular extraction of repurpose chisels on the thermal conductivity, because the samples are cut out in the transversal (TD) and rolling direction (RD), enabling a systematic view of the influence of the carbide morphology.

## 3. Results and Discussion

As already mentioned, the thermal conductivity of the investigated alloys differs due to their chemical composition and heat treatment (Figure 1) [8,9,10]. Therefore, first analysis has to be performed regarding the microstructure of the investigated tool steels. 

### 3.1. Microstructure of the Investigated Alloys

**Figure 2:**
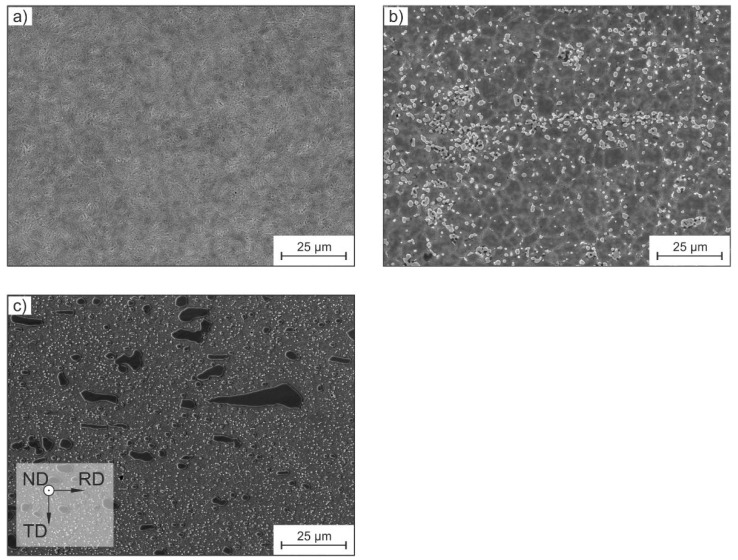
SEM images of (**a**) tool steel L2, (**b**) high-speed steel M2 and (**c**) ledeburitic cold-work steel D2 in the orientation given in the provided axes.

The investigated tool steels showed significant differences in their microstructure and resulting properties (Figure 2). The low-alloyed tool steel L2 showed no hard phases and a fully martensitic microstructure. Regarding the high-speed steel M2, hard phases of type M_2_C, M_6_C and MC were present in a fully martensitic matrix. The cold-work tool steel D2 is characterized by eutectic carbides of the type M_7_C_3_, as shown by Kronenberg et al. [7]. As already mentioned, the larger eutectic carbides are aligned parallel to the primary rolling (RD) direction, which can be seen clearly in the SEM image [7].

### 3.2. Thermophysical Properties as a Function of Temperature

#### 3.2.1. Thermal Conductivity

**Figure 3:**
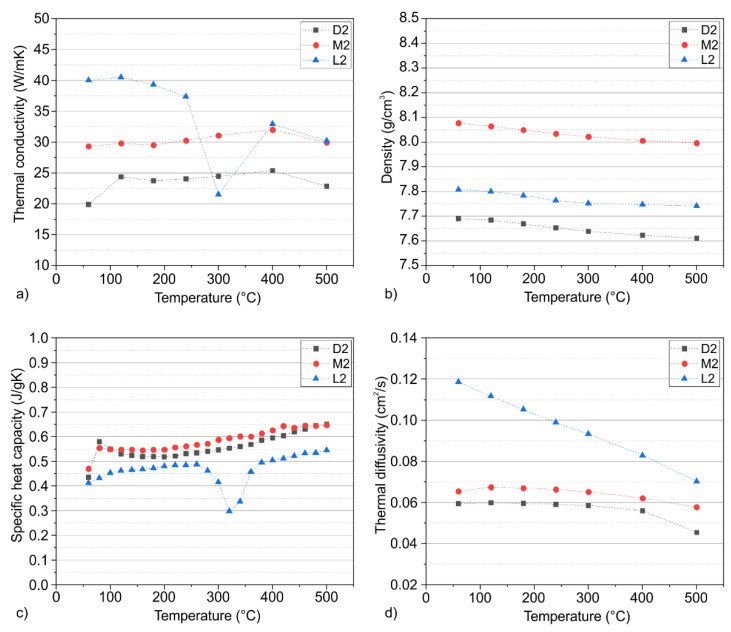
Thermophysical properties of the investigated alloys according to the dynamic measurement method for the calculation of the (**a**) thermal conductivity using the (**b**) density, (**c**) specific heat capacity and (**d**) thermal diffusivity.

Figure 3a presents the thermal conductivity of the tool steels in a temperature range from room temperature up to 500 °C. It is noteworthy that the measurement temperature was lower than the tempering temperature for the M2 and D2 alloy, while it exceeded the tempering temperature of the L2 alloy. As stated in the previous section, a differentiation between the thermal conductivity of the D2 alloy in the rolling (RD) and transversal directions (TD) was not possible using the dynamic measurement method. The material-specific measurements show that the thermal conductivity of the tool steels differed in the whole temperature range. The alloy L2 had the highest conductivity at 60 °C, which is approximately twice as high as the conductivity in the cold-work D2 and over 10 W/mK higher than in the high-speed steel M2. The temperature-dependent slope of the investigated materials show differences, especially when comparing the L2 alloy to the cold-work and high-speed steel. It is well described in the literature that microstructural imperfections and impurities influence thermal conductivity [9,21,23]. These imperfections and impurities are a result of the chemical composition and heat treatment, resulting in a microstructure with carbides and a lattice with a high dislocation density due to the martensitic state. This microstructure assigns an increased number of scattering sources for lattice vibrations and electronic heat carriers, reducing the thermal conductivity, explaining the differences in the thermal conductivity.

A more detailed analysis of the influences and resulting properties is therefore necessary and can be achieved with the help of the individual parameters of the thermal conductivity, namely the density, specific heat capacity and thermal diffusivity, and by consideration of the microstructure.

The temperature-dependent density of the four tool steels is displayed in Figure 3b. The alloy M2 showed the highest density due to the high contents of the heavy elements Mo and W. The tool steel L2 had a similar density to pure Fe through its low concentrations of alloying elements. The Cr-alloyed steels showed a lower density due to the formation of M_7_C_3_ carbides, which have a lower density than pure Fe [24]. Only small differences of the temperature dependence of the alloys were detectable, due to their similar matrix microstructure.

Figure 3c shows the specific heat capacity as a function of the temperature. The trends present show an increase with rising temperature due to the excitement of lattice vibrations. The L2 tool steel was characterized by the lowest values across all studied materials; the specific heat capacity indicates the scattering of phonons in martensitic steels [8]. Through the absence of carbides (Figure 2), the phonons are less scattered, which leads to a lower phonon scattering Another effect is the increase in phononic scattering through alloying elements [25]. According to Williams et al. [25,26], alloying elements are scattering sources as a result of mass differences and induced strain fields in the lattice. The lowest matrix alloy concentration in the tool steel L2 leads to less phononic scattering in the matrix, in addition to previously described effects. As a result, the system requires less energy to increase the temperature and internal energy, respectively. The drop at temperatures around 300 °C is due to precipitation effects, which are a result of the single tempering stage at 200 °C (Table 2). Because of the carbide precipitation, the hardness of the matrix decreases, disabling the deployment of the L2 tool steel in applications that exceed process temperatures of 300 °C.

With respect to the two carbide-rich tool steels D2 and M2, a higher specific heat capacity can be detected. Due to the high content of nanometer-sized tempering carbides in high-speed steels, which are finely dispersed in the matrix [27], the scattering of phonons increases and leads to a higher specific heat capacity. By comparison with the cold-work steel D2, the carbides were smaller and evenly distributed in the high-speed steel and coarser in the cold-work steel, giving the alloy D2 a lower specific heat capacity. 

Thermal diffusivity is a central parameter of thermal conductivity and is defined as the time-dependent component. The thermal diffusivity is determined by factors that also influence the electrical resistivity and the specific heat capacity, because it reflects both electronic and phononic contributions and therefore scattering of those components. By contrasting Figure 3a,d, it can be stated that the thermal diffusivity governs the overall trend of the thermal conductivity.

Considering the thermal diffusivity, the tool steel L2 with the single-phase martensitic matrix showed the fastest heat transfer. As already mentioned, carbides act as impurity sources, thus scattering phonons and reducing the thermal conductivity and diffusivity [9,10,23,28]. Due to this relationship, the alloy L2 also featured the highest thermal conductivity and thermal diffusivity (Figure 2 and Figure 3d). The decrease in the thermal diffusivity results from increased phonon–phonon and phonon–electron scattering, hindering the heat transfer.

The M2 and D2 steels showed a significantly lower diffusivity at low temperatures due to increased scattering by carbides. In contrast to the alloy L2, the thermal diffusivity of the tool steels M2 and D2 was only slightly temperature-dependent. This result is due to the opposing influences of the decreasing effect of carbides and other scattering sources and enhancing influences of the matrix. It is well known that in high-alloyed matrices, the concentration of free electrons increases with temperature, enabling a higher thermal conductivity and diffusivity of the matrix [9,21,29]. It is also well reported in the literature that Cr reduces the concentration of free elements due to enhanced scattering of conduction electrons in the spin-up conduction channel [30]. Nevertheless, the tempering of martensitic steels will increase the thermal diffusivity of the matrix, which occurs especially at higher temperatures [9]. This effect results from the precipitation of tempering carbides, reducing the scattering due to less C and Cr in the matrix. At higher temperatures, the scattering effects dominate due to the interaction of thermally activated phonons and electrons, thus decreasing the diffusivity even more.

#### 3.2.2. Electrical Conductivity and Electronic Thermal Conductivity

To overcome the limitations in the extraction of laser-flash specimens from the chisels, an approach was used based on the electrical conductivity and the Wiedemann–Franz–Lorenz law. Thanks to the more accommodating sample geometry of the specimens for the determination of the electrical resistivity and the Seebeck coefficient, it was possible to measure the influence of the carbide orientation on these properties. This effectively enables a quantitative analysis of the thermal conductivity by means of the electronic part of the thermal conductivity. It is worth mentioning that both the resistivity and the electronic thermal conductivity are proportional to the number of conduction electrons in a metal [21,31].

**Figure 4:**
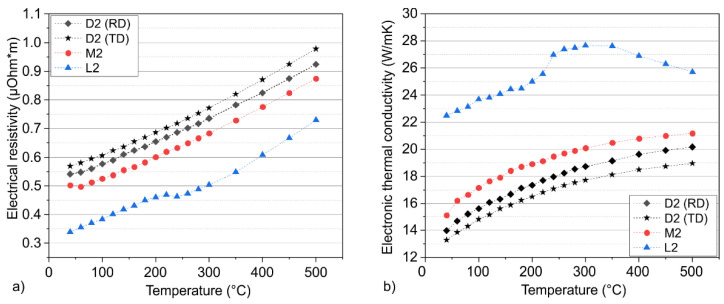
Electrical properties as a function of temperature, regarding the (**a**) resistivity and (**b**) electronic thermal conductivity.

The electrical resistivity is displayed in Figure 4a. As with the thermal conductivity, the alloy L2 had the highest electrical conductivity, which is the reciprocal value of the electrical resistivity. The M2 and D2 tool steels showed a lower electric conductivity. This is due to the influence of Cr on the electronic conduction behavior that was already mentioned [30]. When considering the temperature dependence, it is noticeable that the electrical resistivity increases, which is in unison with Matthiessens’ rule because of the scattering of electrons with the thermally activated phonons with rising temperature [31]. The temperature-dependent slope was similar for all alloys. Nevertheless, the L2 alloy showed a drop in the resistivity, which could be due to the precipitation of C, leading to a less strained martensite. Such a process is expected to decrease the electron–phonon scattering in the matrix, resulting from fewer defects in the lattice which are able to increase the electrical and thermal conductivity.

Of special interest is the influence of the carbide orientation of the D2 tool steel on electrical resistivity. It can be seen that the electronic thermal conductivity of the parallel extracted samples is higher than the conductivity of the samples that have been extracted perpendicularly. This behavior enables a comparison of these two conditions. A detailed view can be achieved by the aid of the electronic part of the thermal conductivity, according to Equation (4).

Regarding the electronic part of the thermal conductivity, the alloy L2 showed the substantially highest values (Figure 4b). The non-linearity at 240 °C is also present in the electronic thermal conductivity, due to the relationship with the resistivity (Equation (4)). Regarding the TD and RD chisels, some differences are noticeable, whereas the temperature dependent slope stays the same. The chisels in the RD were characterized by an increased electronic heat flux compared to the TD-extracted chisels. This is due to the mean free matrix path length in the matrix, which influences the movement of the heat carriers, and therefore the thermal conductivity, significantly in heat-treatable steels [24]. Due to the parallel orientation of the carbides, the matrix free path length is significantly longer, resulting in an increased thermal conductivity, measurable through the electrical resistivity and therefore electronic thermal conductivity.

Therefore, the chisel should be extracted parallel to the rolling direction and carbide orientation in order to increase heat dissipation from the cutting edge and reduce the process temperatures during woodturning due to the increased heat flux. Besides thermal conductivity, other microstructural influences would make a parallel extraction preferable. Arguably, the impact energy will increase with a parallel extraction, because the crack has to pass for a longer distance along the carbides [32,33]. Besides this, Berns et al. [32,33] showed in different studies that an increased wear resistance for the here-investigated tool steel D2 can be achieved if the mean free matrix path length is decreased. Because of the parallel alignment, the carbides are oriented perpendicular to the cutting direction, ensuring an increased wear resistance.

## 4. Conclusions

The current work describes a repurposing strategy implemented in the tool industry, where a worn-out machine circular knife was used in order to extract chisel blanks. These chisels were extracted parallel and perpendicular to the primary rolling direction, which resulted in a parallel and perpendicular carbide orientation of the chisels, respectively. Due to this, different properties were expected: the thermal conductivity was of special interest because of the expected high temperatures that occur during woodturning. Therefore, the thermal conductivity using the dynamic measurement method was measured, where the density, specific heat capacity and the thermal diffusivity were determined separately. Each parameter was therefore investigated as they vary with the measurement temperature, enabling a closer look at each factor individually. Unfortunately, it was not possible to extract samples parallel and perpendicular to the carbide direction out of the chisels for the laser-flash measurements. Therefore, an approach to calculate the electronic contribution to the thermal conductivity using the temperature-dependent measurement of the electrical resistivity and the Seebeck coefficient was used. In this way, the influence of the carbide alignment became measurable. By comparing the chisel material with the material of two conventional chisels, the thermal conductivity of the repurposed chisel was taken into perspective. It was possible to investigate the four tool-steels independently and in a temperature-dependent manner. All parameters were identified as temperature-dependent, making a detailed knowledge of the process temperature indispensable. Nevertheless, it was possible to discover the influences on the thermal conductivity for all investigated materials. The carbide precipitation was systematically identified as the governing factor on the scattering of heat carriers, namely phonons and electrons, reducing the thermal conductivity significantly. This behavior was measurable based on the thermal and electrical conductivity, respectively. Regarding the electrical thermal conductivity, it was possible to detect an influence of the carbide orientation on the thermal conductivity. Because of the free matrix path length, the heat carriers are able to dissipate the energy better from the cutting edge, indicating that the chisel extraction should be carried out parallel to the carbide orientation.

Nevertheless, further studies regarding the wear resistance and the impact on energy consumption should be performed in future, enabling a final decision of the performance of re-purposed chisels.

In particular, the following observations could be made:

(i) Thermal conductivity depends on the microstructure of a material, which depends on the chemical composition and heat treatment.
(ii) Due to the measurement temperature above the annealing temperature, the microstructure of an alloy changes, which is measurable in the thermophysical properties.
(iii) The thermophysical properties are also dependent on the carbide morphology.
(iv) Therefore, the removal of the repurposed chisel parallel to the rolling direction and, accordingly, also to the carbide orientation is to be preferred.

## Figures and Tables

**Table 1:** Chemical composition of the investigated alloys.

Alloy	C	Si	Mn	Cr	Mo	W	V	Fe
L2	0.78	0.23	0.58	0.29	-	-	0.12	Bal.
M2	0.79	0.33	0.34	3.84	4.66	6.28	1.82	Bal.
D2	1.52	0.35	0.43	11.44	0.68	-	0.73	Bal.

**Table 2:** Heat treatment of the investigated alloys.

Material	L2	D2	M2
Hardening	850°/6 min/Oil	1055 °C/30 min/Chill hardening to 70°	1210 °C/15 min/Furnace cooling
Tempering	200 °C/70 min/Salt water	3 × 530 °C/480 min/Furnace cooling	3 × 530 °C/120 min/Furnace cooling

## Data Availability

The raw/processed data required to reproduce these findings cannot be shared at this time as the data also form part of an ongoing study.
